# Characterization of cytoplasmic cyclin D1 as a marker of invasiveness in cancer

**DOI:** 10.18632/oncotarget.8876

**Published:** 2016-04-20

**Authors:** Noel P. Fusté, Esmeralda Castelblanco, Isidre Felip, Maria Santacana, Rita Fernández-Hernández, Sònia Gatius, Neus Pedraza, Judit Pallarés, Tània Cemeli, Joan Valls, Marc Tarres, Francisco Ferrezuelo, Xavier Dolcet, Xavier Matias-Guiu, Eloi Garí

**Affiliations:** ^1^ Department of Cell Cycle, Lleida, Catalonia, Spain; ^2^ Department of Oncological Pathology, Lleida, Catalonia, Spain; ^3^ Department of Biostatistics and Epidemiology Unit of the Institut de Recerca Biomèdica de Lleida (IRBLLEIDA), Lleida, Catalonia, Spain; ^4^ Department of Basic Medical Sciences at University of Lleida, Lleida, Catalonia, Spain; ^5^ Department of Pathology and Molecular Genetics at Hospital Universitari Arnau de Vilanova, Lleida, Catalonia, Spain

**Keywords:** cyclin D1, tissue array, cell invasion, metastasis, Pathology Section

## Abstract

Cyclin D1 (Ccnd1) is a proto-oncogen amplified in many different cancers and nuclear accumulation of Ccnd1 is a characteristic of tumor cells. Ccnd1 activates the transcription of a large set of genes involved in cell cycle progress and proliferation. However, Ccnd1 also targets cytoplasmic proteins involved in the regulation of cell migration and invasion. In this work, we have analyzed by immunohistochemistry the localization of Ccnd1 in endometrial, breast, prostate and colon carcinomas with different types of invasion. The number of cells displaying membranous or cytoplasmic Ccnd1 was significantly higher in peripheral cells than in inner cells in both collective and pushing invasion patterns of endometrial carcinoma, and in collective invasion pattern of colon carcinoma. Also, the cytoplasmic localization of Ccnd1 was higher when tumors infiltrated as single cells, budding or small clusters of cells. To evaluate cytoplasmic function of cyclin D1, we have built a variant (Ccnd1-CAAX) that remains attached to the cell membrane therefore sequestering this cyclin in the cytoplasm. Tumor cells harboring Ccnd1-CAAX showed high levels of invasiveness and metastatic potential compared to those containing the wild type allele of Ccnd1. However, Ccnd1-CAAX expression did not alter proliferative rates of tumor cells. We hypothesize that the role of Ccnd1 in the cytoplasm is mainly associated with the invasive capability of tumor cells. Moreover, we propose that subcellular localization of Ccnd1 is an interesting guideline to measure cancer outcome.

## INTRODUCTION

Cyclin D1 (Ccnd1) is among the 10 proto-oncogenes most frequently amplified in cancer [1]. Indeed, Ccnd1 was first characterized by studies of gene amplification as the gene affected by a chromosome inversion in parathyroid adenoma (PRAD1) and by a translocation t(11:14) in B-Cell Lymphomas (BCL-1) [2]. Alternatively, Ccnd1 was also isolated from a human glioblastoma library in a yeast genetic selection using G1-cyclins depleted cells [3]. The importance of Ccnd1 as an oncogene has been extensively reported. For instance, overexpression of Ccnd1 in the mammary gland is sufficient for the induction of mammary carcinoma [4]. This cyclin is a regulatory subunit of the cyclin-dependent kinases Cdk4/6 that are positive regulators of cell proliferation [5][6][7]. The Ccnd1·Cdk4 complex phosphorylates the transcriptional repressor pRB releasing the E2F-dependent transcription required for S phase entry [8]. Alternatively, Ccnd1 regulates transcription of a different set of genes in a Cdk-independent manner [9]. In both cases, the transcriptional changes induced by Ccnd1 accumulation in the nucleus trigger cell transformation [10].

Ccnd1 has been also associated with tumor invasion and metastasis in clinical studies. For instance, overexpression of Ccnd1 is connected with metastatic prostate cancer to bone [11]. Moreover, knock down of Ccnd1 in xenografted lung adenocarcinoma cells abrogates its metastatic potential [12]. Consistently, it is also established that Ccnd1 enhances cell migration and invasion as Ccnd1^−/−^ cells show a reduction of their potential of invasiveness [13][14]. Interestingly, different works suggest that Ccnd1 is not restricted to the nucleus but is also associated to the cytoplasmic membrane, where it can activate cytoplasmic targets involved in cell invasive potential [15][16][17][18][19]. Cytosolic localization of Ccnd1 has been described in different tumors. For instance, an elevated expression of cytoplasmic Ccnd1 has been detected by IHC in lymph node metastases originated from prostate cancer and is associated with poor survival [20]. However, the possible correlation between cytoplasmic Ccnd1 localization in neoplastic tissues and its ability to enhance invasion and metastasis has not been analyzed yet. In this work, we show that Ccnd1 displays an asymmetric pattern of localization in different cancers, being more cytoplasmic and membranous in the peripheral and invasive regions of the neoplastic tissues. We have mimicked this feature in the cells by using a membrane-attached variant of Ccnd1. We have observed that the sequestration of Ccnd1 in the membrane enhances cell invasion and metastasis without affecting cell proliferation. In the same way as the nuclear localization of Ccnd1 is a signal of proliferation in the IHC analyses, we hypothesize that cytoplasmic localization could be a marker of invasiveness.

## RESULTS

### Cytoplasmic-membranous Ccnd1 expression in human tumor tissue sections

**Figure 1:**
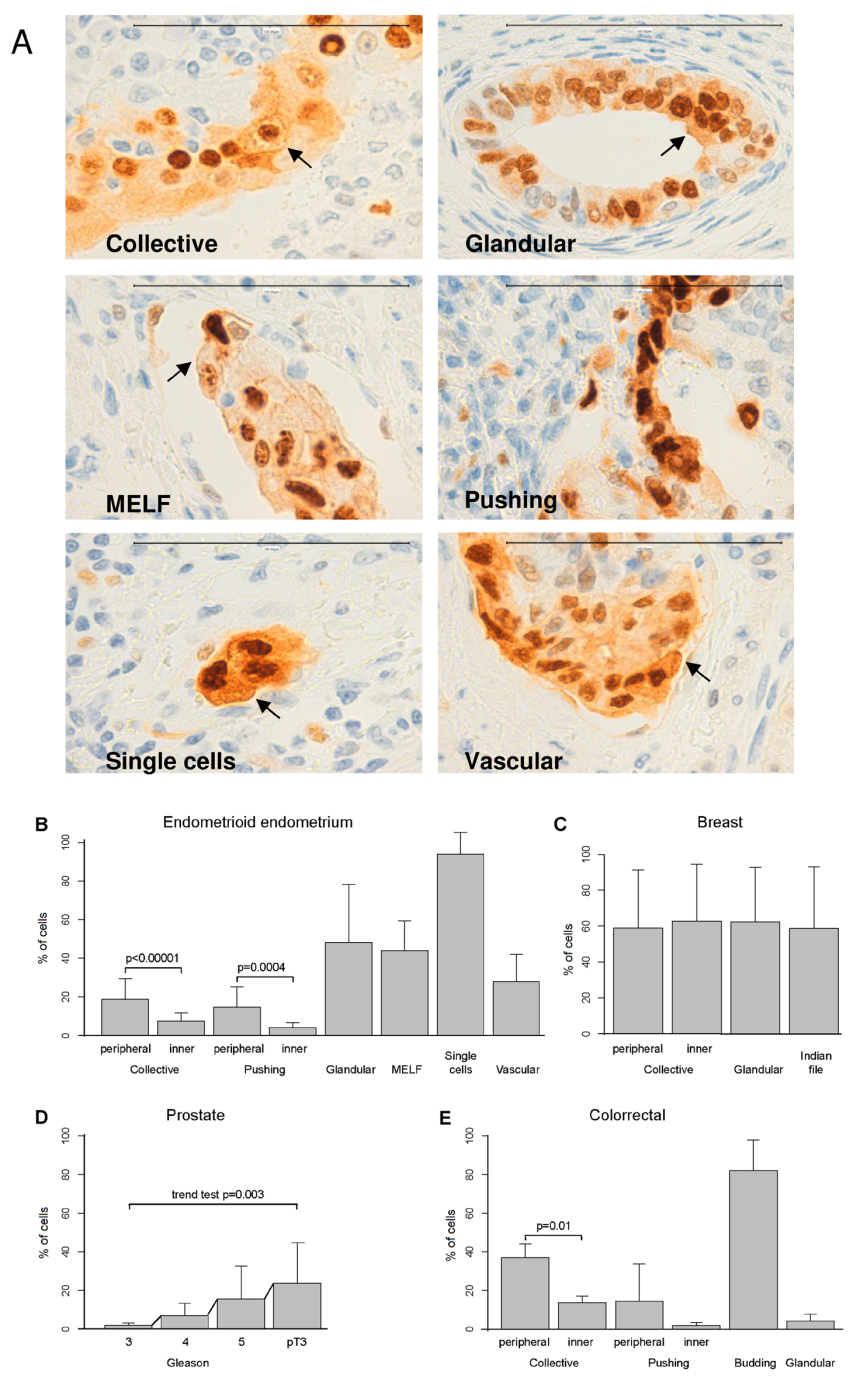
Membranous-cytoplasmic Ccnd1 expression at the invasive front is higher in peripheral cells, in large invasive cell clusters or in specific types of invasion **A.** Representative images showing Ccnd1 expression in endometrioid carcinomas of the endometrium (100μm bar). Different types of invasion are considered (collective, pushing, MELF, glandular, single cells/small cluster of cells, and vascular). Arrows indicate Ccnd1 stain in the membrane. Evaluation of the differences in membranous-cytoplasmic Ccnd1 expression among the different types of invasion in endometrioid endometrial carcinomas **B.**, ductal breast carcinoma **C.**, prostatic carcinoma according to Gleason grade or invasion beyond the prostate (pT3) **D.** and colonic carcinoma **E.** Bars represent mean percentages of positivity and segments one standard deviation. Significant differences between selected pairs are shown with their corresponding p-value, as computed with the linear mixed models. For prostate, *p*-value to evaluate the increasing trend is shown.

To evaluate Ccnd1 localization, we performed IHC analysis of Ccnd1 on different tumoral types. First, we analyzed 55 samples of endometrioid endometrial carcinoma displaying different types of invasion (collective, pushing, glandular, MELF, single cell/small cell clusters, and blood vessel invasion; see Table 1). As we show in Figure 1A, the different types of invasion showed differential patterns of Ccnd1 localization. Cytoplasmic staining seems reticular and not diffuse, and staining in the plasma membrane of certain cells is also observed. According with previous works showing that Ccnd1 is localized in cell membranes [16], we have referred the non-nuclear Ccnd1 as cytoplasmic-membranous localization. Ccnd1 cytoplasmic and membranous expression in collective and pushing patterns of endometrial samples was significantly higher in peripheral cells in comparison with the inner cells (*P* = < 0.00001 and *P* = 0.0004 respectively; Figure 1B). Single cell/small cell cluster, MELF and glandular patterns had the highest Ccnd1 cytoplasmic-membranous expression of all invasion types.

**Table 1:** Definition of the different types of invasion evaluated in the tumours

Invasive Type	ECET^1^	BAC^2^	PAC^3^	CAC^4^
**Collective invasion** Groups of cells invade the peritumoral stroma while maintaining cell-cell contacts.	x	x		x
**Single-cells/single cluster of cells** In the absence of cell-cell adhesion, tumour cells invade as single cells or small cell clusters.	x			
**Budding** Presence of individual cells and small clusters of tumour cells at the invasive front of carcinomas.				x
**MELF** The microcystic, elongated and fragmented (MELF) pattern include the presence of small dilated glands lined by cuboidal or flattened cells with eosinophylic cytoplasm.	x			
**Invasive glands** Invasive glands often have a slit-like appearance.	x	x		x
**Vascular invasion** When tumour cells are noted within or attached to the wall of the vascular space lined by flattened endothelial cells.	x			
**Pushing border invasion** Characterized by a cohesive tumour growth with well-delineated but infiltrating borders and “pushing” margins.	x			x
**Indian-file** The tumour cells spread between the collagen bundles in a single line.		x		
**Gleason 3, 4, 5**			x	
**pT3** Tumour extends beyond the prostate			x	

ECET^1^, Endometrial carcinoma of endometrioid type; BAC^2^, Breast adenocarcinoma; PAC^3^, Prostate adenocarcinoma, CAC^4^, Colorectal adenocarcinoma.

In breast adenocarcinoma, cytoplasmic-membranous Ccnd1 protein expression was evaluated in 50 samples displaying different types of invasion (collective, glandular, indian-file). All invasion types showed high expression of cytoplasmic-membranous Ccnd1 but no differences between peripheral and inner cells in the collective invasion pattern (*P* = 0.18) (Figure 1C; see also Supplementary Figure 1A).

In prostatic adenocarcinoma, cytoplasmic-membranous Ccnd1 protein expression was evaluated in 50 samples, with different types of Gleason grade (3,4,5). Cytoplasmic-membranous Ccnd1 expression increased in parallel with the Gleason grade and, the higher expression occurred in pT3, that is, when tumor extends beyond the prostate (Figure 1D, trend test *P* = 0.003; see also Supplementary Figure 1C).

In colon adenocarcinoma, cytoplasmic-membranous Ccnd1 protein expression was evaluated in 50 samples, with different types of invasion (collective, pushing, budding, glandular). In the collective pattern, cytoplasmic-membranous Ccnd1 expression was significantly higher in peripheral cells in comparison with inner cells (*P* = 0.01). In the pushing pattern, the difference between peripheral and inner cells was not statistically significant (*P* = 0.15). The budding pattern had the highest cytoplasmic-membranous Ccnd1 expression of all invasion types. Interestingly, the expression of Ccnd1 in the cytoplasm and membrane of glandular cells was very low (Figure 1E; see also Supplementary Figure 1B).

Our results show that cytoplasmic-membranous staining for CcndD1 is weaker than nuclear, and a clear membrane signal is only observed in a small fraction of tissue cells. Probably, this result is not uncommon considering that the localization of Ccnd1 in the membrane of cultured cells was also detected only in a fraction of cells [16]. Three hours after seeding on fibronectin, mouse-embryonic fibroblasts and tumor-endometrial cells showed Ccnd1 in the membrane of spreading cells (Supplementary Figure 2A). MFE cells reveal slightly membrane co-localization of Ccnd1 with RalA (Supplementary Figure 2B). The presence of Ccnd1 only in the membrane of spreading cells agrees with the role of Ccnd1·Cdk4 in the regulation of Rho and Ral GTPases activity during adhesion and migration processes [14].

Since membranous-cytoplasmic accumulation of Ccnd1 was seen at the periphery of nests in collective and pushing invasion patterns of endometrial carcinoma samples, but also in correlation with Gleason grade, and pT3 in prostatic cancer, we selected endometrial and prostatic cancer as models to further validate the role of Ccnd1 in invasion.

### The addition of a farnesylation motif to Ccnd1 enhances its localization to the membranes

**Figure 2:**
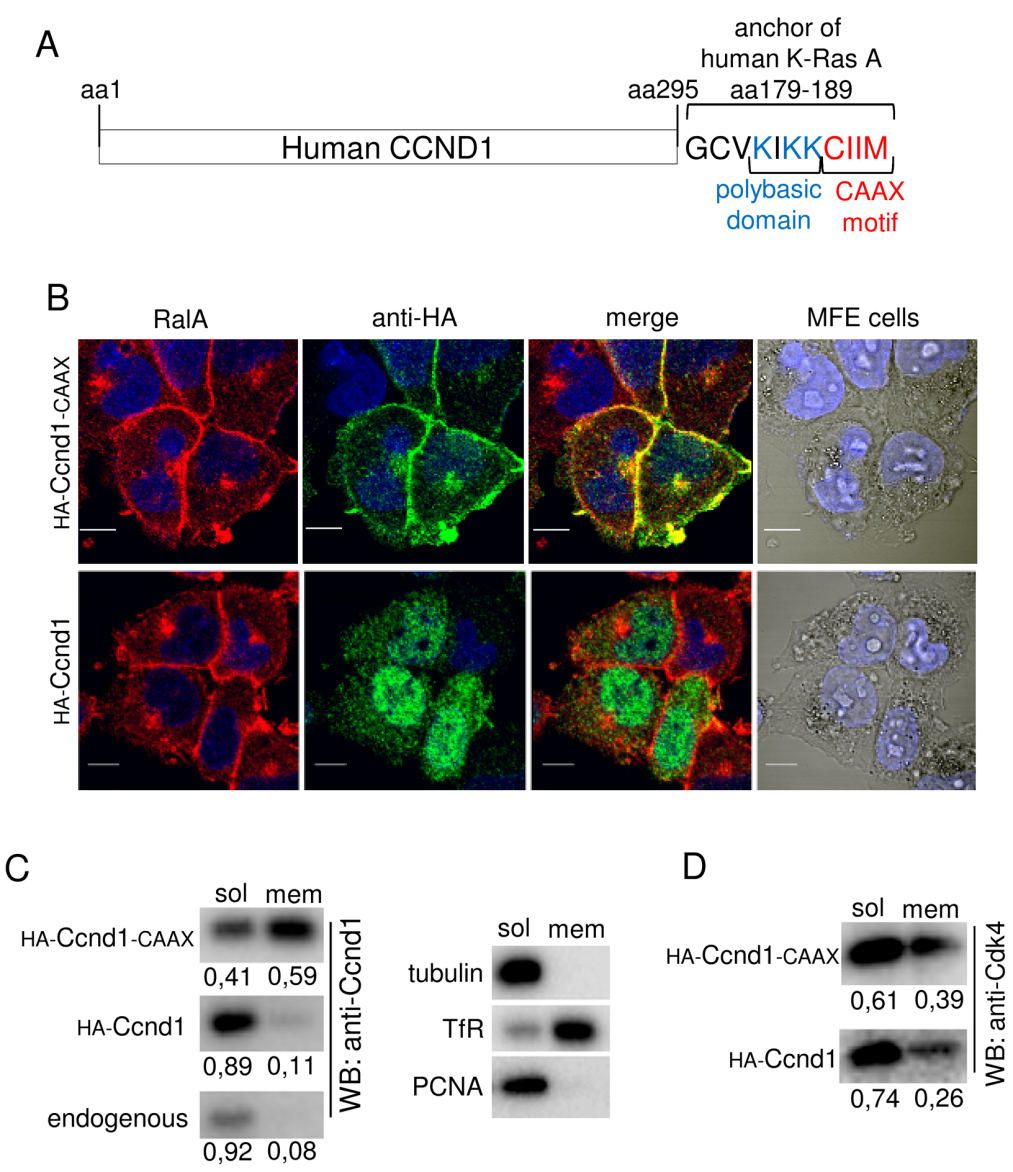
Ccnd1-CAAX is localized in cell membranes of tumor cells **A.** Schematic representation of C-terminal fusion of the anchor domain of human K-Ras with Ccnd1 protein. **B.** MFE cells infected with HA-Ccnd1 or HA-Ccnd1-CAAX or an empty vector were fixed in 4% paraformaldehyde and permeabilized with 0.2% triton-100X. Images were acquired by confocal microscopy (10μm bar). Nuclei were stained with Hoescht (blue). The antibodies used were anti-HA (rat monoclonal 3F10, green) and anti-RalA (mouse monoclonal, red). **C.** Ishikawa cells infected with HA-Ccnd1 or HA-Ccnd1-CAAX were submitted to subcellular fractionation (see “Materials and Methods”). Fractions were analyzed by immunoblotting to detect Ccnd1 in soluble and membrane fractions. Quantification of Ccnd1 levels are shown at the bottom of the panel. Tubulin as a cytosol marker, Transferrin Receptor as a membrane marker and PCNA as a nucleoplasm marker were used to control fractionation. **D.** Cdk4 distribution in soluble and membrane fractions from the experiment in C. Quantification of Cdk4 levels are shown at the bottom of the panel.

We have previously described that Ccnd1·Cdk4 binds to Rgl2 that is a GEF of the Ral GTPases [18]. We hypothesized that Ccnd1·Cdk4 promotes Ral activation, and consequently cell invasion, through the regulation of Rgl2. For Ral activation, Rgl2 has to be recruited to the membrane by the GTPase Ras. An Rgl2 variant containing the membrane-anchor motif of K-Ras promotes constitutive activation of Ral GTPase [21]. Then, to test whether the forced association of Ccnd1 in the membrane could induce invasion of tumor cells, we have built a Ccnd1 with the same membrane-anchor motif of K-Ras fused at the C-terminus. This anchor motif contains a polybasic domain that drives the protein to the negatively charged membrane and a CAAX motif with a cysteine residue to attach a farnesyl tail [22] (Figure 2A). The endometrial cells were infected with lentiviruses containing either HA-Ccnd1 or HA-Ccnd1-CAAX alleles and were grown in complete medium. After five days, cells were fixed and processed for immunofluorescence using the rat 3F10 anti-HA antibody to visualize the localization of the exogenous Ccnd1. We observed that the wild type allele was localized in both the nucleus and the cytoplasm whereas the Ccnd1-CAAX allele was mostly localized at the membrane and cytoplasm (Figure 2B). Moreover, we determined that only Ccnd1-CAAX widely co-localizes in the membrane with the GTPase RalA that naturally contains a farnesylation motif and is localized in the membrane. These results indicate that the addition of K-Ras farnesylation motif to Ccnd1 can efficiently target it to the membrane.

To confirm this result, we analyzed subcellular localization of Ccnd1 and Ccnd1-CAAX alleles by cell fractionation. Endometrial cells (Ishikawa) were infected with virus containing Ccnd1 or Ccnd1-CAAX and processed for cell fractionation using a Subcellular Protein Fractionation kit (Thermo Scientific-Pierce; 78840). We obtained a soluble fraction containing cytosolic and nuclear soluble proteins and a membrane fraction as indicated by the reference proteins (Figure 2C). We observed that the presence of Ccnd1-CAAX was extensively enriched in the membrane fraction (Figure 2C). Interestingly, the endogenous and exogenous wild type Ccnd1 exhibited also a reduced portion of the protein in the membrane fraction in accordance with previous works suggesting that Ccnd1 interacts with membrane-associated proteins as filaminA or RalA [16][18]. In these works, it has been suggested that cytoplasmic functions of Ccnd1 are Cdk4-dependent. For that reason, we have tested whether Cdk4 was also present in the membrane fraction (Figure 2D). As expected, a portion of Cdk4 was in the membrane and it was also enriched after Ccnd1-CAAX expression. All these results indicate that the expression of Ccnd1-CAAX promotes the accumulation of Ccnd1-Cdk4 complexes in the membrane.

### The sequestration of Ccnd1 in the membrane increases the invasiveness of tumor cells but does not change proliferation in those cells

**Figure 3:**
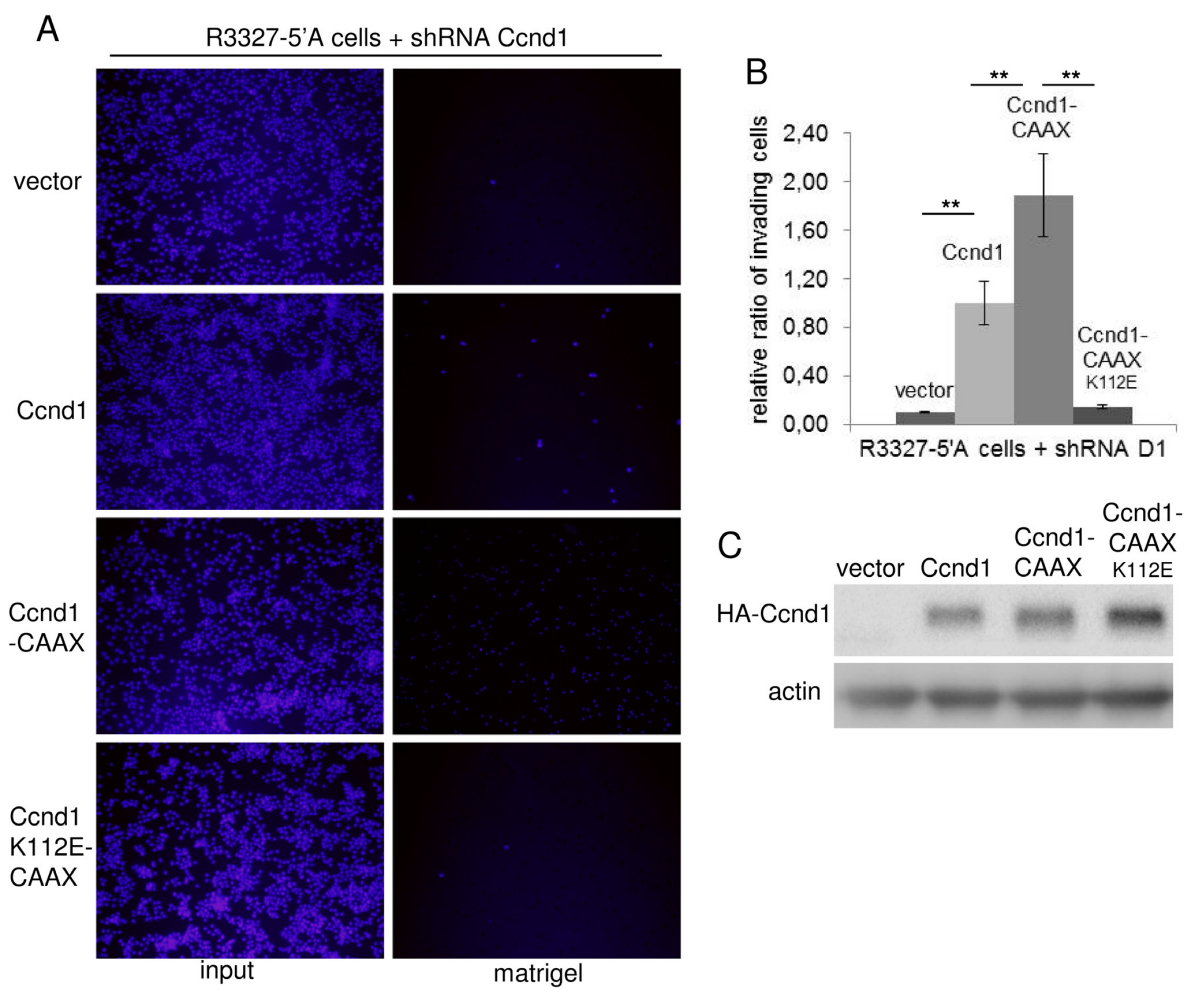
The membrane-associated Ccnd1 enhances invasiveness of tumor cells **A.** R3327-5′A rat cells were infected with interference shRNA against rat Ccnd1 (shD1, Sigma). Cells expressing the shRNA were further infected with human Ccnd1, Ccnd1-CAAX, Ccnd1_K112E_-CAAX or with an empty lentiviral vector. For invasion assays, 5×10^4^ co-infected cells were seeded in 24-well transwell filters previously coated with matrigel, and allowed to invade for 24 hours. After this time all cells were fixed and stained with Hoescht -input-. Then, the remaining cells at the bottom of the filter were washed and Matrigel-embedded cells were counted -matrigel-. **B.** Relative values from the experiment in A are expressed in mean ± sem. Data are from at least three independent experiments. Significance values were determined by one way ANOVA and Holm Τ-statistic post-test (**p < 0.01). **C.** Immunoblot showing the expression of HA-Ccnd1 in the co-infected cells. Actin was used as a loading control.

Our study of Ccnd1 localization in cancer tissues suggested that the cytoplasmic localization of Ccnd1 may be related to the invasiveness of tumor cells (see above). It has also been reported that Ccnd1 could have cytoplasmic substrates involved in the regulation of cell migration and invasion [16][18]. For these reasons, we sought to analyze whether the membrane-associated allele of Ccnd1 could significantly increase the invasiveness. We infected R3327-5′A rat tumor cells that had endogenous Ccnd1 downregulated by RNA interference with lentiviruses containing human HA-Ccnd1, HA-Ccnd1-CAAX or HA-Ccnd1_K112E_-CAAX or empty vector. After five days growing in complete medium, those cells were processed to reveal “*in vitro*” their invasive potential. We observed that the expression of Ccnd1-CAAX significantly induced the invasiveness of the R3327-5′A cells in comparison with the expression of the wild type allele or the empty vector (Figure 3A and 3B). It has been previously reported that Ccnd1 enhanced cell invasiveness by a Cdk4-dependent mechanism [14]. In accordance with that result, we also remarked that the expression of the inactive allele Ccnd1_K112E_-CAAX was unable to promote cell invasion. All these results suggest that the sequestration of an active complex Ccnd1-Cdk4 in the membrane increases the invasiveness of tumor cells.

It has been reported that overexpression of Ccnd1 reduces doubling-cell time and increases clonogenic and anchorage-independent growth in transformed cells [23][24]. Moreover, Ccnd1 alleles or mutants that enhance nuclear accumulation increase clonogenic growth capability over the wild type allele, even in non-transformed cells [25][26]. Then, we questioned whether the membrane-associated allele of Ccnd1 could estimulate cell proliferation. To test this hypothesis, MFE cells were infected with lentiviruses containing HA-Ccnd1 or HA-Ccnd1-CAAX or empty vector. After growing, those cells were handled to determine the proliferation rate and clonogenic and anchorage-independent growth. The expression of Ccnd1-CAAX did not significantly change the proliferation rate of MFE cells compared to the wild type Ccnd1 (Supplementary Figure 3A). Also we noted that neither the clonogenic nor non-adherent growth was modfified by the expression of Ccnd1-CAAX in MFE cells (Supplementary Figure 3B and 3C). These results suggest that sequestration of Ccnd1 in the membrane does not modify any form of growth in tumor cells.

### The attachment of Ccnd1 in the membrane promotes RalGTPase activation

**Figure 4:**
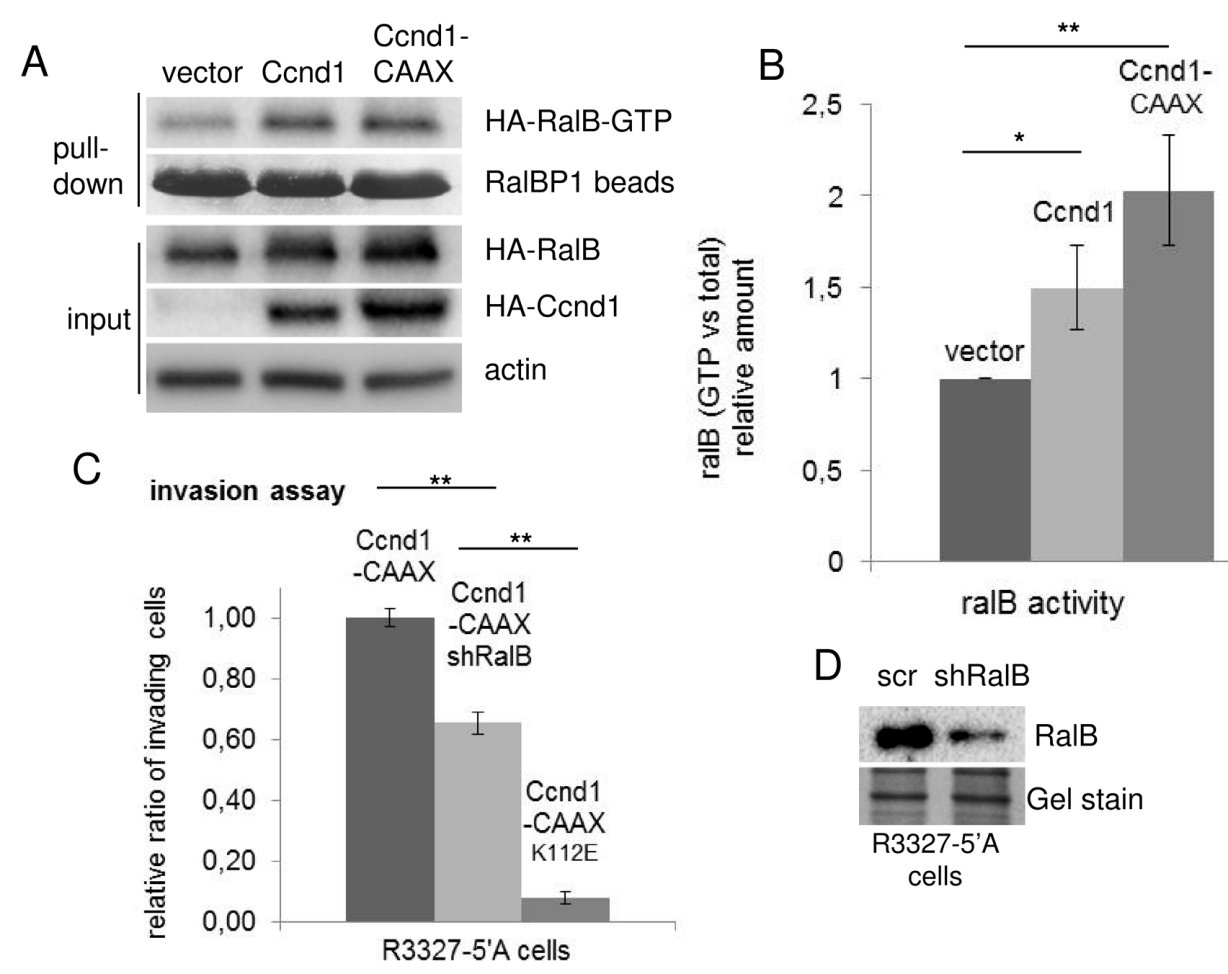
The membrane-associated Ccnd1 promotes Ral-GTPase activation **A.** HEK293T cells were co-transfected with HA-RalB and either HA-Ccnd1, or HA-Ccnd1-CAAX or an empty vector as a negative control. Twenty-four hours after transfection, active RalB-GTP was affinity purified with RalBP-beads from cell lysates and detected by immunoblotting with anti-HA antibody. HA-D1, HA-RalB and HA-RalB-GTP amount from a representative experiment is shown. RalBP-beads and actin were used as loading controls. **B.** The experiment was independently repeated four times. Relative mean values ± sem for the HA-RalB-GTP/ total HA-RalB ratio are plotted. Significance values were determined by one way ANOVA and Holm Τ-statistic post-test (*p < 0.05; **p < 0.01). **C.** R3327-5′A rat cells were infected with interference shRNA against RalB (shRalB) or with scramble (scr) as a control. Relative invasion values are expressed in mean ± sem. Data are from three independent experiments. Significance values were determined by one way ANOVA and Holm Τ-statistic post-test (**p < 0.01). **D.** Immunoblot showing the levels of expression of RalB in (C). Gel stain was used as a loading control.

In a previous report we described that cyclin D1 enhanced cell motility through the activation of RalGTPases [18]. Then, we expected that the attachment of Ccnd1 to the membranes would ameliorate the activation of RalGTPases and consequently the efficiency of cell invasion. To analyze this possibility, we have co-expressed HA-Ccnd1 or HA-Ccnd1-CAAX with HA-RalB in HEK293T cells and then, we determined the levels of the HA-RalB loaded with GTP in those cells. The expression of Ccnd1-CAAX enhanced RalB activity better than the expression of Ccnd1, even though the increment in RalB activity was not statistically significant (Figure 4A and 4B). Since the membrane attachment of Ccnd1 was able to appreciably improve the invasiveness of tumor cells, we tested whether the improvement in invasion was RalB-dependent. The down-regulation of RalB by shRNA in R3327-5′A cells expressing Ccnd1-CAAX partially reduced the invasion capacity of these cells (Figure 4C and 4D). Our data suggest that membrane-associated Ccnd1 promotes invasion in some extent through Ral activation, but it may have other interactors or substrates than Ral GTPases to completely explain the role of Ccnd1 in the membrane.

### The presence of Ccnd1 attached to the membrane of tumor cells stimulates the tumor invasion and metastasis

**Figure 5:**
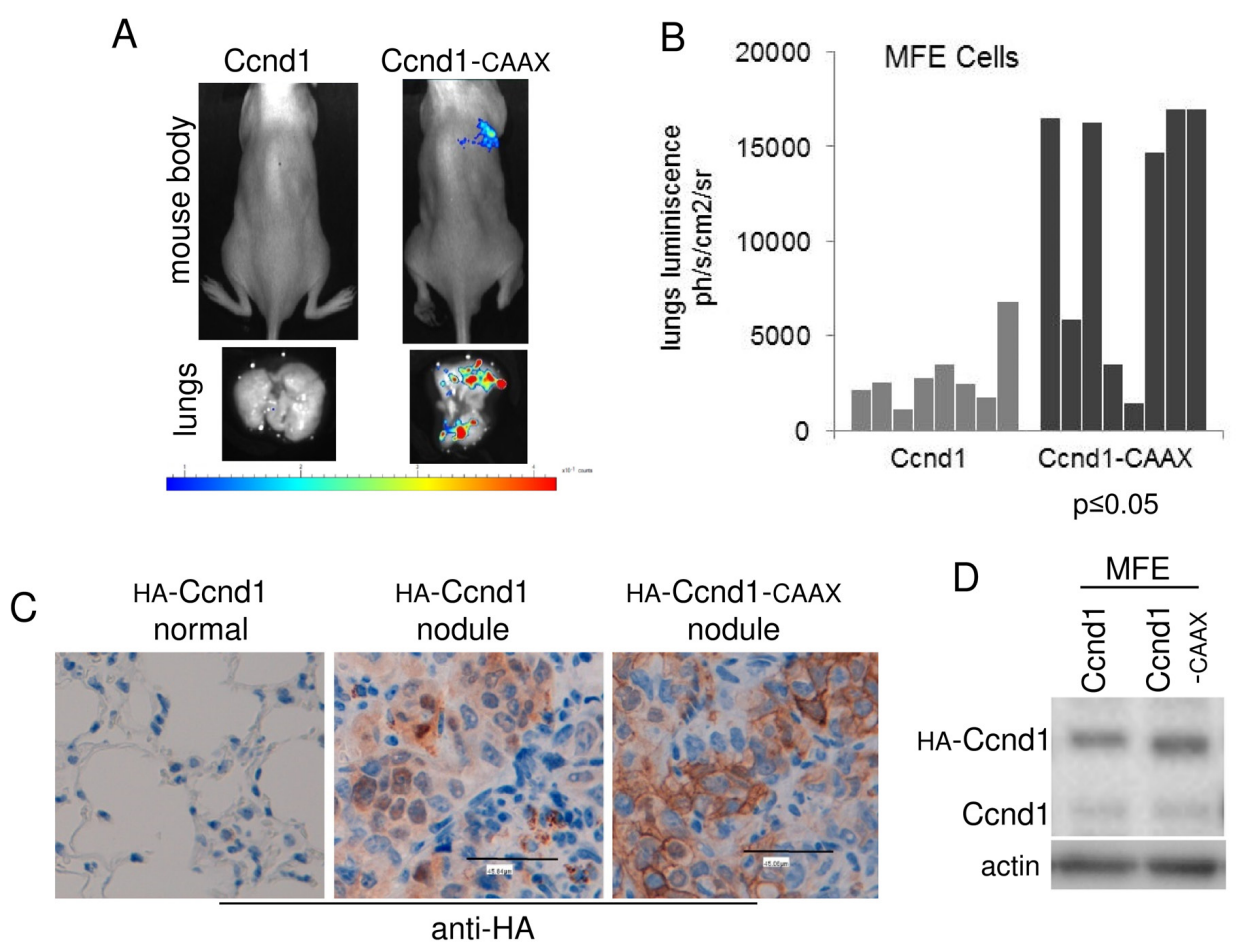
The membrane-associated Ccnd1 enhances lung metastatic activity of endometrial tumor cells **A.** MFE cells stably expressing luciferase were used for metastasis assay. Cells harboring Ccnd1 or Ccnd1-CAAX were injected in SCID-hairless mice. Four weeks after injection, animals expressing Ccnd1-CAAX showed lung metastasis. Representative images of the body luminescence (top) and lungs luminiscence (bottom) are shown. **B.** The number of lung nodules from eight animals in A was inferred from luminescence and represented. Significance values were determined by a Mann-Whitney test. **C.** Representative HA-immunohistochemistry images of lung nodules are shown. **D.** MFE cells expressed similar amounts of Ccnd1 or Ccnd1-CAAX. A protein extract was obtained from the cells before injection to the mice, and the amount of Ccnd1 was analyzed by immunoblot. Actin was used as a loading control.

Ccnd1 has been described as an inductor of metastasis in mice and in clinical studies [27][12][11]. To measure the relevance of the membrane-attachment of Ccnd1 in the induction of metastasis “*in vivo*”, we injected MFE cells expressing Ccnd1-CAAX or Ccnd1 directly to the bloodstream of 8-weeks-old nude mice. Animals were sacrificed four weeks after injection, their lungs examined for metastatic growth and the presence of nodules in the lungs was calculated (Figure 5A and 5B). We observed that the expression of Ccnd1-CAAX significantly stimulated MFE-dependent metastasis (six out of the eight animals exhibited apparent nodules). After four weeks, only one animal manifested metastasis when the wild type allele of Ccnd1 was expressed in MFE cells. We confirmed the presence of the MFE cells in the nodules by revealing HA signal in the tissues, HA-Ccnd1-CAAX was detected in the membrane of cells (Figure 5C). Note that the abundance of metastases in the samples expressing Ccnd1-CAAX *versus* Ccnd1 was not due to differences in the expression levels (Figure 5D). Similar results were obtained using prostatic cancer cells (Supplementary Figure 4). Overall, this result suggests that the presence of Ccnd1 in the cell membrane of tumor cells may be a marker of poor prognosis. This hypothesis is consistent with the data about cancer tissues analyzed in the first part of this paper.

## DISCUSSION

The molecular mechanisms involved in both individual and collective tumor cell invasion are not fully understood. From the histopathological viewpoint, there are many different patterns of invasion, which frequently coexist in different areas of the same tumor. Some tumor types show specific patterns of invasion, which are not seen in other types of cancer. This phenomenon is clearly represented by endometrial cancer, which has a peculiar type of invasion (MELF, characterized by microcystic, elongated, and fragmented tumor cells in a fibromyxoid stroma) that is very infrequent in other tumor types. A relevant question is whether there are generic elements altered in all those patterns of invasion [28][29]. In this study, we have found an increase of the membranous-cytoplasmic Ccnd1 expression in cells from specific invasion patterns (single cell/small cell clusters, buds, glandular) in which tumor cells are in contact with the adjacent stroma. More interestingly, increased membranous-cytoplasmic Ccnd1 expression was seen in peripheral cells in comparison with inner cells, whenever cancer cells infiltrate the stroma as large cell masses (collective or pushing patterns). Moreover, membranous-cytoplasmic Ccnd1 expression was seen in peculiar forms of invasion, such as the MELF pattern. Considering all these data, we hypothesize that the presence of membranous-cytoplasmic Ccnd1 is a generic indicator of invasive phenotype.

We have also shown that forced attachment of Ccnd1 to the membrane (caused by Ccdn1-CAAX expression) induces tumor cell invasiveness and metastasis in tumor cells through a RalB-dependent mechanism. Considering all the data cited, we propose that the localization of Ccnd1 in the cytoplasm do not merely mean an appeasement of proliferation but could be a device to induce migration and invasion. Certainly, the role of Ccnd1 in the regulation of cell migration and invasion is also dependent on nuclear functions. Li et al, 2006 showed that the transcriptional repression of the cytoskeleton regulators ROCK II and TSP-1 exerted by Ccnd1 is required for efficient fibroblasts migration. Then, our proposal should not be categorically understood like that Ccnd1 shows a dichotomy as an oncogene, proliferation *versus* invasion, that can be unveiled depending on the subcellular localization, nucleus *versus* cytoplasm, respectively. By immunohistochemistry, Ccnd1 shows a diverse pattern of localizations in cancer tissues and cytoplasmic accumulation does not mean nuclear exclusion and *vice versa* ([30]; our results). Thus, we can envisage that when a detectable amount of Ccnd1 moves to the cytoplasm may be suggestive of an increment in the invasive potential of the cells.

The knowledge of the subcellular pattern of Ccnd1 may be interesting to determine the proliferative and invasive ability of specific tumors. It is well accepted that Ccnd1 nuclear accumulation is indicative that the tumor has a significant mitotic activity. For instance, cytoplasmic localization of Ccnd1 is observed in low-grade prostate carcinomas with reduced Ki-67 positivity whereas nuclear Ccnd1 is associated with high degree and elevated Ki-67 [30]. Conversely, cytoplasmic expression of Ccnd1 has not been taken into account as an indicator of tumor status other than the non-proliferative state. Nevertheless, single cell and MELF pattern in endometrial carcinoma, budding in colon cancer and pT3 tumors of prostatic carcinomas each of them exhibiting membranous-cytoplasmic Ccnd1 expression, have been associated with aggressiveness [31][32][33][34][35][36][37]. Then, we propose that cytoplasmic Ccnd1 could be indicative of tumor cells with a high invasive potential.

## MATERIALS AND METHODS

### Tumor tissue samples

Tumor samples, classified following the most recent WHO criteria, included 55 cases of endometrioid endometrial carcinoma, 50 cases of colorectal adenocarcinoma, 50 cases of breast carcinoma, and 50 cases of prostatic carcinoma. They were obtained from the Surgical Pathology files of Hospital Universitari Arnau de Vilanova, and IRBLLEIDA Biobank in Lleida, Spain. A specific informed consent was obtained from each patient in accordance with the protocols approved by the Local Ethical Committee. Tumors had been fixed in formalin and embedded in paraffin. Selected sections and paraffin blocks containing the invasive front of the tumors into the adjacent tissue were retrieved. Several specific types of tumor invasion, depending of tumor site, were evaluated (Table 1). In prostatic cancer, Gleason grade (3, 4 and 5) was taken as indirect indicator of invasion, and pT3 prostatic cancers were selected as the subgroup exhibiting the most prominent invasive features, by infiltrating peri-prostatic tissues.

### Cell culture

MFE and Ishikawa (IK) cells are from endometrial adenocarcinoma. R3327-5′ rat prostate-tumor cells were kindly provided by M. Hendrix. HEK293T cells were obtained from the American Type Culture Collection. Cells were maintained at 37°C in a 5% CO_2_ incubator, and grown in Dulbecco's modified Eagle's medium (DMEM) supplemented with 10% Fetal Bovine Serum (FBS), 100 μg/ml penicillin/streptomycin and 2 mM glutamine. Transient transfection of vectors was performed with Lipofectamine 2000 (Invitrogen) according to manufacturer's instructions. For lentivirus production, HEK293T cells were transfected with lentiviral expression vectors, envelope plasmid pVSV.G, and packaging plasmid pHR'82ΔR at a 2:1:1 ratio.

### Expression vectors

Human CCND1 wild type and CCND1_K112E_ inactive allele were used to obtain an N-terminal 3xHA fusion under the UBI promoter in a lentiviral vector derived from pDSL (Invitrogen) or under the CMV promoter in a pCDNA3 vector. In these constructs the 3′ end of the K-Ras ORF containing the CAAX motif (GGC TGT GTG AAA ATT AAA AAA TGC ATT ATA ATG TAA) was fused to the 3′ end of the CCND1 ORF. For RalB activity assay we performed an N-terminal 3xHA fusion to human RalB (image 3880116) and cloned in a pCDNA3 vector. Details of all constructs are available upon request. The RALB MISSION shRNA TRCN0000072957 and the CCND1 MISSION shRNA TRCN0000026883, both cloned in a pLKO.1-puro, were obtained from Sigma.

### Immunofluorescence

Briefly, cells were quickly washed in PBS and fixed in 4% paraformaldehyde for 15 min at room temperature. Fixed MFE cells or fibroblasts were permeabilized with 0.2% triton-100X for 3 min at room temperature, and blocked with 3% BSA. Primary antibodies were combined with adequate Alexa488 and/or Alexa594-labeled secondary antibodies (Molecular Probes) in PBS with 0.3% BSA. Nuclei were stained with Hoechst (Sigma). Images were acquired using 60X objective in an Olympus FV1000 confocal system. The antibodies used were anti-HA (rat monoclonal 3F10, green), anti-Ccnd1 (rabbit monoclonal EP12 Dako, green) and anti-RalA (mouse monoclonal, red).

### Protein fractionation and immunoblot

Protein fractionation was performed with the Subcellular Protein Fractionation kit for cultured cells (Thermo Scientific-Pierce; 78840). Soluble fraction corresponds to a mixture of cytosolic and nuclear soluble fractions described in the supplier's instructions.

For immunoblot, protein samples were resolved by SDS-PAGE, transferred to PVDF membranes (Millipore), and incubated with primary antibodies anti-Ccnd1 (monoclonal DCS-6, BD Pharmigen), anti-Cdk4 (polyclonal C-22, sc-260), anti-HA (rat monoclonal 3F10, Roche), anti-TfR (monoclonal H68.4, Invitrogen), anti-RalB (rabbit polyclonal, Cell Signaling), anti-PCNA (monoclonal PC10, Abcam) and anti-tubulin (monoclonal B-5-1-2 Sigma). Appropriate peroxidase-linked secondary antibodies (GE Healthcare UK Ltd) were detected using the chemiluminescent HRP substrate Immobilon Western (Millipore). Chemiluminescence was recorded with a ChemiDoc-MP imaging system (BioRad).

### Cell invasion assay

We performed cell invasion assays in 6.5-mm filters of 8.0 pore size (Transwell, Corning). Filters were coated with Matrigel (reduced-factors, BD Biosciences) in the upper side. Then, cells (5×10^4^) were seeded in the bottom side of the filter for four hours to allow their attachment. Afterwards, filters were loaded with DMEM 10% serum and incubated in 24-well plates containing serum-free medium for 24 hours. Under these conditions, some cells migrate from the bottom to the upper side of the filter invading the Matrigel. All cells were fixed and stained with Hoescht. Remaining cells at the bottom of the filter were removed and Matrigel-embedded cells were counted.

### Ral pull-down assay

The Ral activation was analyzed by measuring the GTP-bound form of Ral. The assays were performed by using RalBP1 agarose (Upstate, cat# 14-415) according to the manufacturer's instructions. Cell lysates were obtained from one 100 mm plate from transfected HEK293T cells. The lysis buffer used was 50 mM Tris pH 7.5, 200 mM NaCl, 2.5 mM MgCl_2_, 2.5 mM DTT, 1% Triton and protease and phosphatase inhibitors. 0.6 ml of cell lysate was incubated with 10μg of RalBP1 beads during 30 min at 4°C and, after several washes, agarose beads were resuspended in 2x Laemmli buffer. Samples were separated by SDS-PAGE, transferred to PVDF membranes, and immunoblotted.

### Metastasis assay

The procedure performed in this study followed the National Institutes of Health Guidelines for the Care and Use of Laboratory Animals, and was compliant with the guidelines of our Institution (UdL). Immunodeficient female SCID hr/hr mice (8-week-old) were maintained in Specific Pathogen Free (SPF) conditions, and were inoculated with 5×10^5^ MFE cells by retroorbital intravenous injection. Animals were sacrificed four weeks afterwards. For the metastasis assays injecting R3327-5′A cells, we have used fewer cells (2.5×10^4^) and analyzed the lungs one week after injection, as those cells are very aggressive inducing metastasis.

### Immunohistochemistry

Paraffin blocks of human tumor tissue samples were sectioned at a thickness of 3μm, dried for 1 hour at 65°C before deparaffinization, rehydration, and epitope retrieval in the Pre- Treatment Module, PT LINK (Dako, Glostrup, Denmark) at 95°C for 20 minutes in 50Å~ Tris/EDTA buffer, pH 9. Before staining the sections, endogenous peroxidase was blocked. Samples were subjected to IHC for Ccnd1 (1:25, EP12, DAKO), visualized with the EnVision FLEX Detection Kit (Dako, Glostrup, Denmark) using diaminobenzidine chromogen as a substrate. Slides were counterstained with hematoxylin. Negative controls were obtained without the addition of the primary antibody. The immunohistochemical analysis was conducted by pathologist and a researcher to ensure a pre-established histological criteria. IHC staining was assessed using ImageJ formal manual count method, 1,47v by Wayne Rasband (National Institutes of Health, USA), by analyzing tumor cells in a mean number of 5 invasive foci per case. Results indicate proportion of cells positively stained in the cytoplasm and membrane of selected fields (approximately 50 high resolution fields, X40). In the collective and pushing patterns, we assessed the difference in positively stained cytoplasm and membrane between peripheral and inner cells. Table 1 presents all invasion types evaluated in each of the different tumours studied.

Regarding mouse tissues, lung tissue samples were fixed with PFA. Blocks were sectioned at a thickness of 3 μm and dried for 1 h at 65°C, before being dewaxed in xylene and rehydrated through a graded ethanol series, then washed with PBS. Antigen retrieval was performed by heat treatment in a pressure cooker for 2 min in EDTA (pH 8.9). Before staining the sections, endogenous peroxidase was blocked. The antibody used was anti-HA 12CA5 mouse monoclonal (ascitic fluid). After incubation, the reaction was visualized with the EnVision Detection Kit (Dako), using diaminobenzidine chromogen as a substrate. Sections were counterstained with hematoxylin.

### Statistical analysis

For IHC data, mean and standard deviation were computed to assess the positivity observed in the cytoplasm for each tumoral type (endometrium, breast, prostate and colon) when comparing different invasion types or histological grades. Linear mixed models were used to evaluate the significance of the differences, using a random effect to take into account the samples corresponding to the same individual. All analyses were made using R, setting the threshold for significance at 5% (alfa = 0.05).

For cell data, comparisons among groups were made by one way ANOVA and Holm Τ-statistic post-test (*p < 0.05, **p < 0.01, ns no significant).

For animal studies we have used the Ene 3.0: Program to calculate sample size. This software was developed by the Department of Applied Statistics of Autonomous University of Barcelona and is distributed by GlaxoSmithKline. No specific method of randomization was used but the group allocation was done randomly. Significance values were determined by Mann-Whitney U-Test Calculator (significance values 0.05 and two-tailed test).

## SUPPLEMENTARY MATERIALS FIGURES


