# Supplementary material for: Characterization of cytoplasmic cyclin D1 as a marker of invasiveness in cancer
Source: Oncotarget. 2016 Apr 20;7(19):26979–91. doi: 10.18632/oncotarget.8876 (PMC5053626; doi:10.18632/oncotarget.8876)
Supplement: Supplementary file 1 [file oncotarget-07-26979-s001.pdf]

Characterization of cytoplasmic cyclin D1 as a marker of invasiveness in cancer

Supplementary Material

Clonogenic assay

MFE cells were infected with the indicated lentiviruses and three days after the infection seeded onto 6-well plates at a density of 1×10^3 cells per well. Cells were incubated for 15 days and colonies were stained with crystal violet for 30 minutes and fixed with 4% paraformaldehyde for 5 minutes and maintained in 2 mL PBS. Colonies were scored using the Quantity One program (Colony Counting Quick Guide) and two replicate dishes were counted for each condition.

Colony Formation Assay in soft agar

Three days after infection Ccnd1 or Ccnd1-CAAX-transduced cells were trypsinized and resuspended in 0.3% agar diluted in medium at concentration of 3×10^3 cells/ml. One ml of cell suspension was layered on a 0.6% agar-coated 35 mm culture dish. Dishes were incubated at 37°C with saturating humidity and 5% CO_2 for 15 days. Colonies of three independent experiments were visualized by staining with Mitotracker (MTT). Colonies were counted with Quantity One software (Bio-Rad).

Growth and viability assay.

MFE cells were infected with the indicated lentiviruses. Three days after the infection, cells were plated in 24-well plates at 5×10^3 cells/cm^2. After overnight incubation, cells of two independent experiments were counted in hemocytometer every 24 h. To assay the viability cells were diluted in Trypan Blue dye.

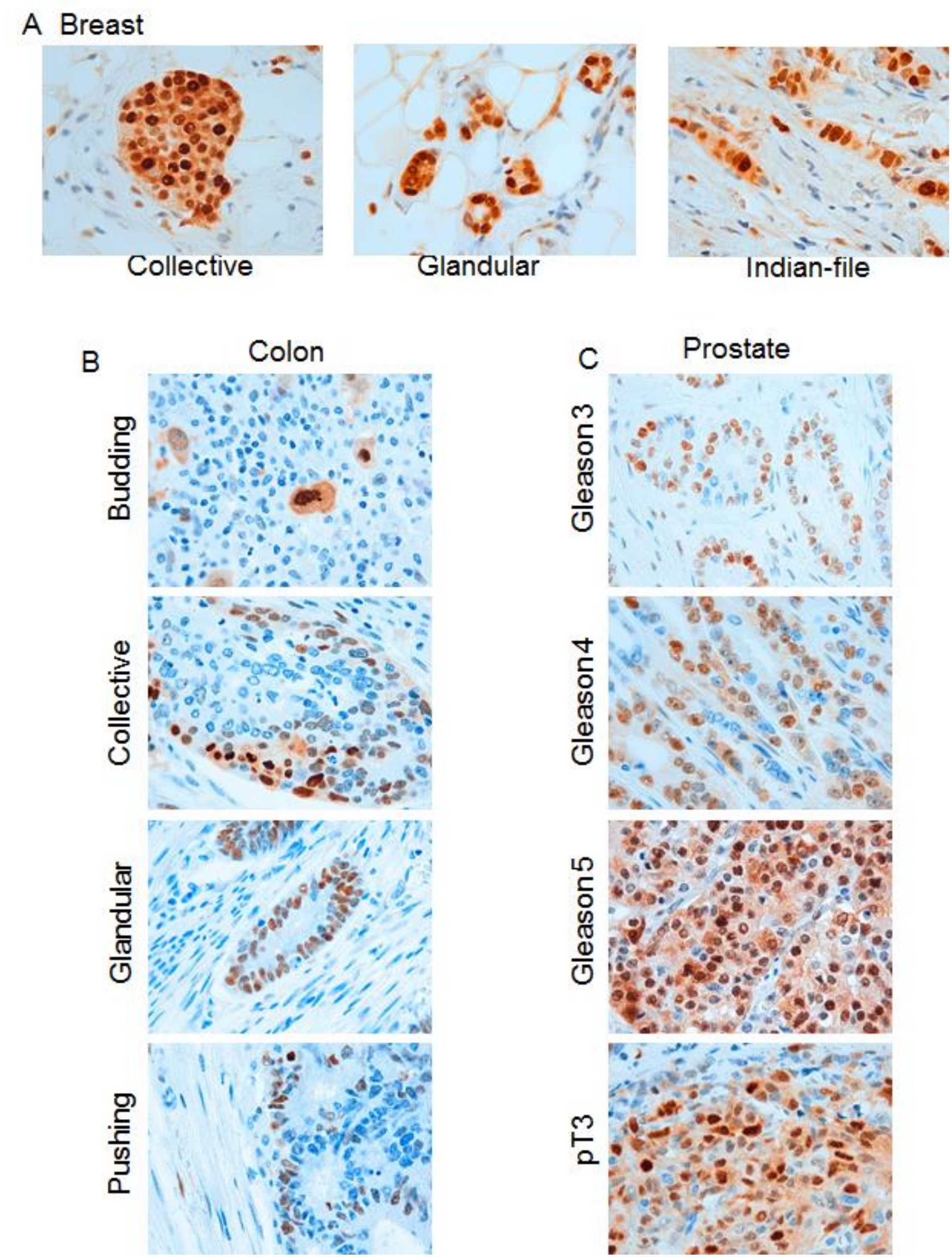

Supplementary Figure S1. Membranous-cytoplasmic Ccnd1 expression at the invasive front is higher in peripheral cells in large invasive cell clusters or in specific types of invasion. Representative images showing Ccnd1 expression at the invasive front in different types of invasion in breast, prostatic and colon carcinomas. See also Figure 1.

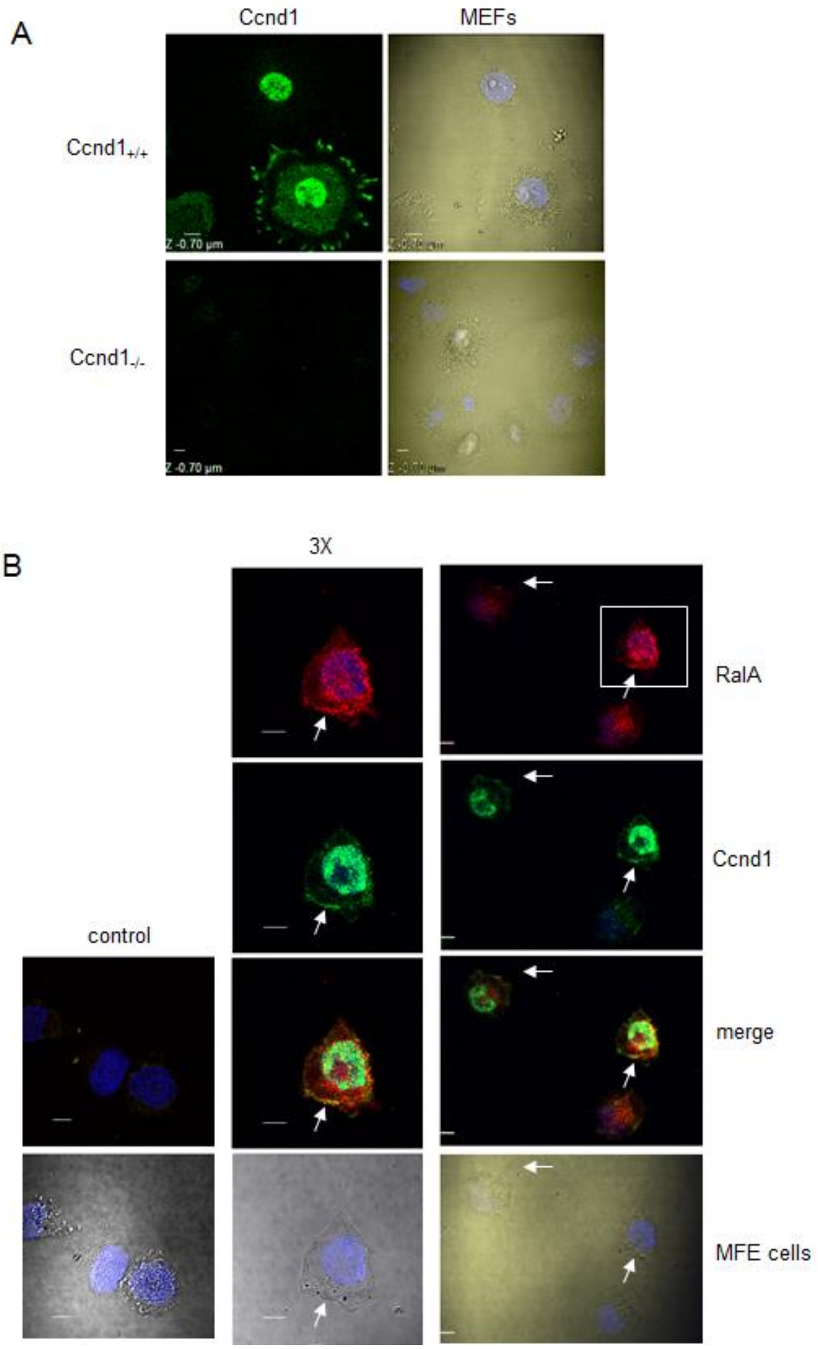

Supplementary Figure S2. **Cyclin D1 is associated to the membranes of fibroblasts and tumor cells during spreading.** (A) Ccnd1−/− and Ccnd1+/+ fibroblasts were fixed in 4% paraformaldehyde and permeabilized with 0.2% Triton X-100. Images were acquired by confocal microscopy (10μm bar). Nuclei were stained with Hoescht (blue). Anti-Ccnd1 (rabbit monoclonal clone EP12) was used. (B) Images of cultured MFE cells were processed as in A (10μm bar). Anti-Ccnd1 (EP12) and anti-RalA (mouse monoclonal) antibodies were used. The square shows one of the cells amplified (3x). Arrows indicate the presence of Ccnd1 in the membrane co-localizing with RalA. Cells incubated only with Alexa488 and/or Alexa594-labeled secondary antibodies were used as a control.

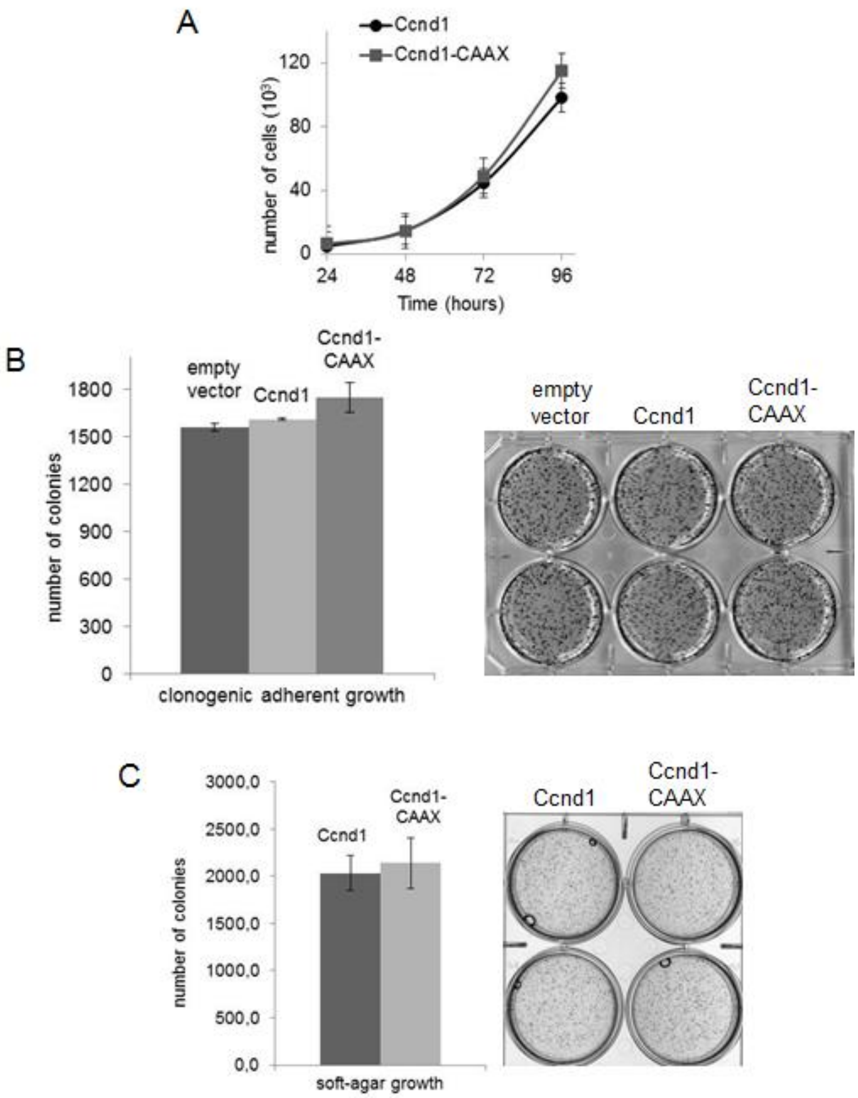

Supplementary Figure S3. The membrane-associated Ccnd1 does not change the proliferation of tumor cells. (A) MFE cells were infected with lentiviruses carrying Ccnd1 or Ccnd1-CAAX. Three days after infection cells were plated at $5x10^3$ cells/cm^2 in 24-well plates and allowed to adhere overnight. Cells were counted every 24 h for 4 days. Growth curves are plotted and each point is the mean ± sd from two independent experiments. No significant differences were detected. (B) MFE cells were infected with lentiviruses carrying the empty vector or Ccnd1 or Ccnd1-CAAX. Three days after infection 10^3 cells were seeded as a single-cell suspension in 6-well plates. Fifteen days after seeding, colonies were stained (right) and results were plotted as mean ± sem (left). No significant differences were detected. (C) MFE cells were infected as in A and three days after infection cells were trypsinized and resuspended in 0.3% agar diluted in medium at a concentration of 3 x 10^3 cells/ml. Fifteen days after plating, colonies were stained (right) and scored as mean ± sem (left). No significant differences were detected.

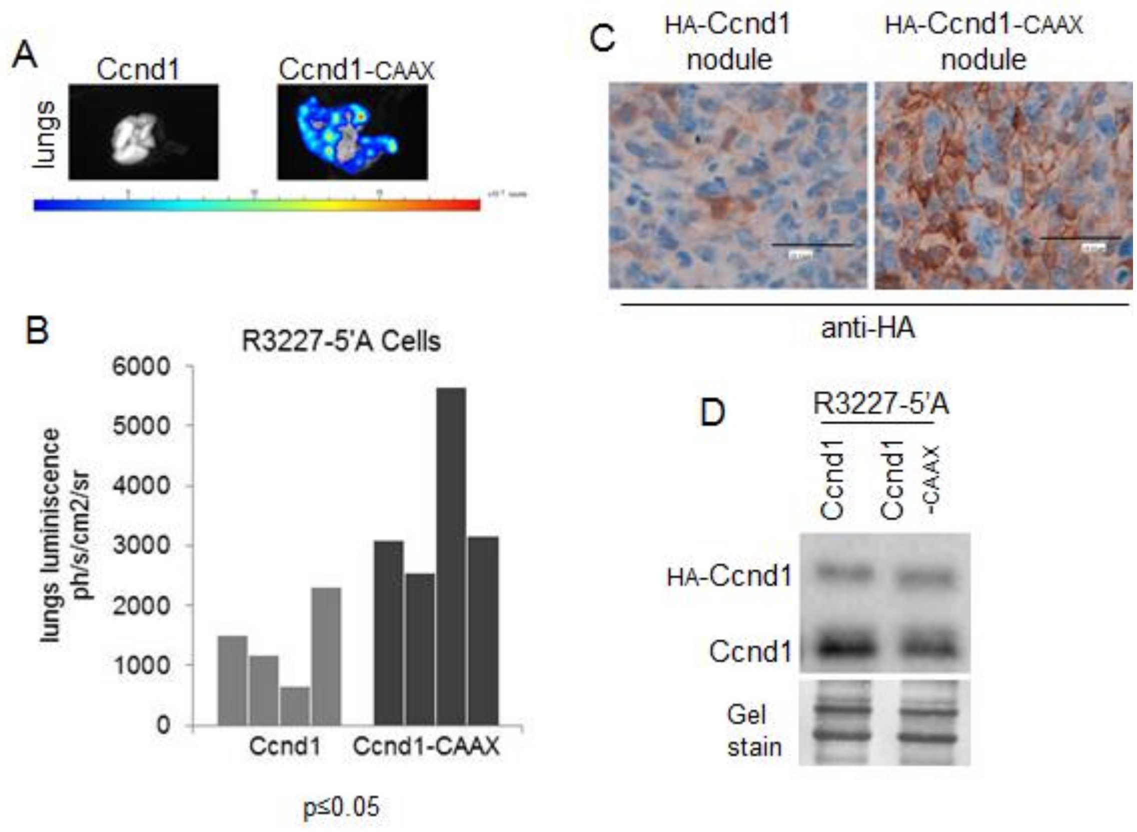

Supplementary Figure S4. **The membrane-associated Ccnd1 enhances lung metastatic activity of prostatic carcinoma cells.** (A) R3227-5'A cells stably expressing luciferase were used for metastasis assay. Only $2{,}5x10^4$ cells harboring Ccnd1 or Ccnd1-CAAX were injected in SCID-hairless mice. One week after injection, animals expressing Ccnd1-CAAX enhanced lung metastasis. A representative image of lungs luminiscence is shown. (B) The number of lung nodules from four animals in A was inferred from luminescence and represented. Significance values were determined by a Mann-Whitney test. (C) Representative HA-immunohistochemistry images of lung nodules are shown. (D) R3327-5'A cells expressed similar amounts of Ccnd1 or Ccnd1-CAAX. A protein extract was obtained from the cells before injection to the mice, and the amount of Ccnd1 was analyzed by immunoblot. Gel stain was used as a loading control.
